# Depression and Anxiety Among Pregnant and Non‐Pregnant Women in Bangladesh: A Cross‐Sectional Study

**DOI:** 10.1002/hsr2.73019

**Published:** 2026-08-09

**Authors:** Md. Rashidul Islam Rifat, Md. Shehab Uddin Ikhtiyer, Foysal Rahman Ovi, Md. Onthor, Tasnia Rahman Shefa, Most. Farjana Yesmin

**Affiliations:** ^1^ Department of Statistics Mawlana Bhashani Science and Technology University Tangail Bangladesh

**Keywords:** anxiety, BDHS, depression, mental health, pregnancy

## Abstract

**Background and Aims:**

Depression and anxiety are common among women of reproductive age, yet nationally representative evidence comparing pregnant and non‐pregnant women in Bangladesh remains limited. This study aimed to estimate the prevalence and severity of depression and anxiety and examine their association with pregnancy status.

**Methods:**

This cross‐sectional study analyzed data from the 2022 Bangladesh Demographic and Health Survey (BDHS), including 20,029 women aged 15–49 years, of whom 1181 were pregnant. Depression and anxiety were assessed using the Patient Health Questionnaire‐9 (PHQ‐9) and Generalized Anxiety Disorder‐7 (GAD‐7). Survey‐weighted Descriptive statistics, chi‐square tests, and hierarchical binary logistic regression analyses were performed to examine associations between pregnancy status and mental health outcomes. Adjusted odds ratios (AORs) with 95% confidence intervals (CIs) were reported. Analyses were conducted using IBM SPSS Statistics version 27.0, with statistical significance set at *p* < 0.05.

**Results:**

Mean depression scores were approximately similar between pregnant and non‐pregnant women (3.40 ± 3.25 vs. 3.34 ± 3.37). Mean anxiety scores were descriptively lower among pregnant women (2.77 ± 3.02 vs. 3.12 ± 3.17). No statistically significant difference was observed in depression severity (*p* = 0.398), whereas anxiety severity differed significantly by pregnancy status (*p* = 0.012). In the fully adjusted hierarchical binary logistic regression model, pregnancy was not significantly associated with depression (AOR = 1.29, 95% CI: 0.97–1.71), but was independently associated with higher odds of anxiety (AOR = 1.41, 95% CI: 1.04–1.92).

**Conclusion:**

Pregnancy was not associated with depression but was independently associated with increased odds of anxiety after adjustment. These findings highlight the importance of accounting for confounding factors and support the integration of routine mental health screening into maternal and reproductive health services. Further longitudinal research is needed to clarify causal relationships.

## 1 Introduction

Depression and anxiety represent critical challenges for global public health, standing as primary drivers of non‐fatal disease burden and disability among women in their reproductive years [1]. The vast majority of this psychiatric burden occurs in low‐ and middle‐income countries (LMICs), where resource constraints and limited infrastructure leave families to shoulder roughly 82% of all cases [2, 3]. These common mental disorders (CMDs) have extensive societal impacts, accounting for nearly 11% of the total disease burden in developing nations and 14% on a global scale, while also sharing a deep, two‐way relationship with physical health issues [3, 4]. Worldwide patterns show that women are impacted far more severely than men, with women facing a 50% higher likelihood of experiencing depressive episodes and twice the risk of developing chronic anxiety conditions [5, 6, 7, 8]. Overall, statistics suggest that approximately one in five women will struggle with a common mental disorder at some point during their lifetime [9]. Pregnancy is one of the periods in a woman's reproductive life where this baseline vulnerability may be most sharply reshaped, for reasons that are both biological and social, and it is this reshaping that motivates the present study.

The first of these pathways is biological. Pregnancy involves rapid shifts in gonadal hormones such as estrogen and progesterone, which act directly on brain circuits and neurotransmitter systems responsible for mood regulation [10, 11]. Layered onto this neuroendocrine shift is a psychological adjustment: the transition into motherhood requires renegotiating self‐image, family role, and identity, alongside anticipatory worry about childbirth and infant health, all of which draw on a woman's emotional coping capacity [11, 12, 13]. Evidence on the net effect of this biological pathway is mixed. Some studies report elevated perinatal depression and anxiety, while others find stable or even lower symptom levels when women have strong supportive environments around them. These findings suggest that biological influences interact with social context rather than acting in isolation. Although these biological mechanisms cannot be directly measured within the BDHS, they provide the rationale for examining pregnancy status as the primary exposure in the present study.

That social context constitutes a second, psychosocial pathway, and in Bangladesh it pulls in two directions at once. On one hand, women of childbearing age frequently face a triple burden of early marriage, immediate expectation to bear children, and relocation into the husband's family home [14, 15], transitions associated with loss of personal autonomy, restricted access to financial resources, and heightened exposure to intimate partner violence, all established risk factors for depression and anxiety [16, 17, 18, 19]. On the other hand, pregnancy occupies a celebrated place in Bengali culture, where an expected child confers elevated social status, increased family attention, and practical help from the extended household [20]. Pregnancy may therefore act as both a source of psychological stress and a period of increased social support, depending on household circumstances. Accordingly, the association between pregnancy and mental health cannot be assumed and requires direct empirical comparison between pregnant and non‐pregnant women.

Testing this, however, depends on the measurement tool being fit for purpose. The Patient Health Questionnaire‐9 (PHQ‐9) and Generalized Anxiety Disorder‐7 (GAD‐7) are widely used standards for screening depression and anxiety and have been validated in Bengali, with acceptable psychometric performance in South Asian populations [21, 22, 23]. These instruments were also used by the 2022 Bangladesh Demographic and Health Survey (BDHS) to assess mental health among women of reproductive age, providing the basis for the present analysis. Both instruments have also been directly applied to pregnant populations across a range of settings and study designs. A large multinational study used PHQ‐9 and GAD‐7 together to assess antenatal mental health in over 7000 pregnant women across middle‐ and high‐income countries [24]. A 2025 Bangladeshi facility‐based study administered Bengali‐adapted versions of both scales to antenatal care attendees, reporting depressive symptoms in 39% and anxiety symptoms in 50% of participants [25]. At much larger scale, a longitudinal study of over 41,000 pregnant women in China used both instruments repeatedly across early, mid, and late pregnancy to track how depressive and anxiety symptoms evolve through gestation [26]. Both instruments, however, were developed and validated in general, non‐pregnant populations, and several of their items, fatigue, sleep disturbance, appetite change, difficulty concentrating, overlap with somatic changes that are normal in a healthy pregnancy. This overlap risks inflating apparent symptom scores among pregnant women independent of any true change in mental state [12, 13]. Rather than treating this as a limitation to be noted only after the fact, we treat it as central to our design: by administering the same instrument to pregnant and non‐pregnant women drawn from the same national sample, the comparative approach at least holds the measurement tool constant across groups, isolating differences attributable to pregnancy status from differences attributable to the scale itself. It does not fully resolve the somatic‐overlap concern, but it is the strongest available check against it in the absence of a pregnancy‐specific validated alternative. Accordingly, comparing pregnant and non‐pregnant women using the same validated screening instruments within a nationally representative sample provides a consistent and methodologically appropriate approach for evaluating whether pregnancy status is associated with depression and anxiety.

Despite the scale of the problem, evidence on depression and anxiety in Bangladesh remains fragmented. Existing studies have relied on small or narrowly drawn samples, university students, clinic attendees, or specific age groups, producing prevalence estimates that vary widely and generalize poorly [27, 28, 29]. Nationally representative data have been especially scarce, in part because Bangladesh has not participated in the WHO's World Mental Health surveys [30]. Where national estimates do exist, they point to a substantial burden: the 2019 Bangladesh National Mental Health Survey found that 18.8% of adult women had a diagnosable mental health disorder, including depression, anxiety, or stress [30], yet that survey could not speak to whether pregnancy itself shifts this risk, since it was not designed for that comparison.

The 2022 BDHS partially addresses this evidence gap by incorporating the PHQ‐9 and GAD‐7 into a nationally representative survey for the first time in Bangladesh [31]. Since its release, the BDHS 2022 mental health module has been successfully utilized in several recent peer‐reviewed studies investigating depression, anxiety, and related mental health outcomes among Bangladeshi women [32, 33, 34, 35], providing additional support for the suitability of these standardized screening instruments in nationally representative epidemiological research. Unlike previous hospital‐ or community‐based studies, the BDHS used standardized data collection procedures and validated Bengali versions of both instruments, enabling robust population‐level comparisons of depression and anxiety between pregnant and non‐pregnant women.

Using data from the 2022 BDHS, this study estimates and compares the prevalence and severity of depression and anxiety among pregnant and non‐pregnant women of reproductive age. In addition, hierarchical multivariable logistic regression models were used to examine whether pregnancy status was independently associated with depression and anxiety after sequential adjustment for key socio‐demographic characteristics, including age, place of residence, educational attainment, household wealth, religion, and marital status. By providing nationally representative evidence, this study aims to improve understanding of maternal mental health in Bangladesh and inform policies and antenatal care strategies for the early identification and management of depression and anxiety.

## 2 Materials and Methods

### 2.1 Study Design and Data Source

This study employed a cross‐sectional design and analyzed data from the 2022 BDHS [31], a nationally representative survey conducted by National Institute of Population Research and Training (NIPORT) in collaboration with ICF International. The survey used a two‐stage stratified cluster sampling method to capture both urban and rural areas across all eight divisions. First, enumeration areas were selected based on the latest census, and then households within these areas were systematically sampled. Women aged 15–49 years in the chosen households were interviewed in person using structured questionnaires by trained data collectors [17].

### 2.2 Study Population and Sample Selection

The BDHS 2022 included 30,078 women of reproductive age [17, 31]. For this analysis, only participants with complete information on pregnancy status, depression, and anxiety were included, resulting in a final sample of 20,029 women, 1181 pregnant and 18,848 non‐pregnant, allowing for a direct comparison of mental health outcomes by pregnancy status.

### 2.3 Measures

Depression was assessed using the PHQ‐9, a validated screening tool consisting of nine items measuring depressive symptoms over the past 2 weeks. Each item is scored on a 4‐point Likert scale ranging from 0 (“not at all”) to 3 (“nearly every day”), yielding a total score between 0 and 27. Scores were categorized as minimal (0–4), mild [5, 6, 7, 8, 9], moderate [10, 11, 12, 13, 14], moderately severe [15, 16, 17, 18, 19], and severe [20, 21, 22, 23, 24, 25, 26, 27] [21]. For logistic regression analysis, a binary variable was created where scores ≥ 10 were classified as “depression present” and scores < 10 as “no depression”.

Anxiety was measured using the GAD‐7 scale, which includes seven items assessing symptoms over the past 2 weeks. Each item is scored from 0 to 3, producing a total score ranging from 0 to 21. Scores were classified as minimal (0–4), mild [5, 6, 7, 8, 9], moderate [10, 11, 12, 13, 14], and severe [15, 16, 17, 18, 19, 20, 21] [23]. For logistic regression, a binary outcome variable was generated, where scores ≥ 10 indicated “anxiety present” and scores < 10 indicated “no anxiety.”

Pregnancy status was determined using the BDHS variable V213 (“Currently pregnant”), based on the question “Are you currently pregnant?”, with responses coded as 1 = “Yes” and 0 = “No or unsure.”

Other covariates included age (categorized in 5‐year groups), place of residence (urban/rural), educational attainment (no education, primary, secondary, higher), household wealth index (poor, middle, rich), religion (Islam/others), and marital status (married/others).

### 2.4 Statistical Analysis

All analyses incorporated individual sampling weights to account for the survey design and to obtain nationally representative estimates. Descriptive statistics were used to summarize participants' characteristics and estimate the prevalence and severity of depression and anxiety. Mean PHQ‐9 and GAD‐7 scores were calculated and descriptively compared between pregnant and non‐pregnant women. The associations between pregnancy status and the severity categories of depression and anxiety were assessed using chi‐square tests.

To examine the association between pregnancy status and mental health outcomes, separate hierarchical multivariable logistic regression models were fitted for depression and anxiety. Pregnancy status was considered the primary exposure variable. Covariates were selected a priori based on previous literature and their potential role as confounders. Variables were entered sequentially in three conceptual blocks. Model 1 included pregnancy status only. Model 2 additionally adjusted for age, place of residence, educational attainment, and household wealth index. Model 3 further included religion and marital status. This modeling approach allowed us to evaluate whether the association between pregnancy status and the outcomes remained consistent after progressively adjusting for potential confounding factors.

Model fit was assessed using the Omnibus Test of Model Coefficients, Hosmer–Lemeshow goodness‐of‐fit test, −2 Log Likelihood, and Cox and Snell and Nagelkerke pseudo‐$R^{2}$ statistics. Results are presented as adjusted odds ratios (AORs) with 95% confidence intervals (CIs). All analyses were performed using IBM SPSS Statistics version 27.0, and statistical significance was set at *p* < 0.05. This study was reported in accordance with the STROBE guideline for cross‐sectional studies.

## 3 Results

### 3.1 Characteristics of the Participants

Table 1 presents the sociodemographic characteristics of the study participants. The largest proportion of women was aged 25–34 years. Most participants resided in rural areas (71.30%) and had completed secondary education (46.90%). Socioeconomic status was relatively evenly distributed across wealth categories. The majority were married (99.30%) and identified as Muslim (90.0%), while 5.9% of respondents reported being currently pregnant.

**Table 1:** Sociodemographic characteristics of study participants.

Variable	Category	Frequency	Percentage
Age group (years)	15–19	1729	8.60
	20–24	3289	16.40
	25–29	3523	17.60
	30–34	3437	17.20
	35–39	3374	16.80
	40–44	2502	12.50
	45–49	2174	10.90
Residence	Urban	5752	28.70
	Rural	14,277	71.30
Education	No education	2642	13.20
	Primary	5206	26.00
	Secondary	9396	46.90
	Higher	2785	13.90
Socioeconomic status	Poor	7798	38.90
	Middle	4124	20.60
	Rich	8107	40.50
Religion	Islam	18,027	90.00
	Others	2002	10.00
Currently pregnant	No or unsure	18,848	94.10
	Yes	1181	5.90
Current marital status	Married	19,897	99.30
	Others	132	0.70

### 3.2 Overall Prevalence and Severity of Depression and Anxiety

Table 2 presents the overall prevalence and severity of depression and anxiety among the participants. The mean depression score was 3.35 ± 3.36, and the mean anxiety score was 3.10 ± 3.17. Most participants reported minimal or mild symptoms for both conditions, indicating generally low levels of clinically significant depression and anxiety in the study population.

**Table 2:** Mental health status of overall respondents (*N* = 20,029).

Measure	Depression	Anxiety
Mean ± SD	3.35±3.36	3.10±3.17
**Severity levels**		
Minimal	14,258 (71.20%)	14,724 (73.50%)
Mild	4784 (23.90%)	4429 (22.10%)
Moderate	739 (3.70%)	690 (3.50%)
Moderately severe	201 (1.00%)	—
Severe	47 (0.20%)	186 (0.90%)
**Binary classification**		
Depression/Anxiety present (Scores ≥ 10)	987 (4.90%)	876 (4.40%)

For depression, 71.20% of participants had minimal symptoms and 23.9% had mild symptoms, while a small proportion experienced moderate (3.70%), moderately severe (1.00%), or severe (0.20%) depression. Based on binary classification, 4.90% of participants were classified as having depression.

Similarly, for anxiety, 73.50% of participants reported minimal symptoms and 22.10% reported mild symptoms. Moderate anxiety was observed in 3.50% of participants, and severe anxiety in 0.90%. Overall, 4.40% of participants were classified as having anxiety.

### 3.3 Descriptive Comparison of Mean Depression and Anxiety Scores by Pregnancy Status

Table 3 presents the descriptive comparison of mean depression and anxiety scores between pregnant and non‐pregnant women. Pregnant women had slightly higher mean depression scores (3.40 ± 3.25) compared to non‐pregnant women (3.34 ± 3.37), although the difference was small.

**Table 3:** Comparison of PHQ and GAD scores by pregnancy status.

Pregnancy status	*N*	PHQ‐9 score (Mean ± SD)	GAD‐7 score (Mean ± SD)
No or unsure	18,848	3.34±3.37	3.12±3.17
Yes	1181	3.40±3.25	2.77±3.02

In contrast, mean anxiety scores were lower among pregnant women (2.77 ± 3.02) than among non‐pregnant women (3.12 ± 3.17), suggesting mean anxiety scores were descriptively lower among pregnant women.

### 3.4 Prevalence and Severity of Depression and Anxiety by Pregnancy Status

#### 3.4.1 Prevalence and Severity of Depression by Pregnancy Status

Table 4 presents the distribution of depression prevalence and severity according to pregnancy status. The prevalence of depression was similar between pregnant and non‐pregnant women. The distribution of depression severity categories was also comparable across both groups. For example, the proportion of minimal depression was 70.50% among pregnant women and 71.30% among non‐pregnant women, while moderate depression was observed in 4.10% and 3.70%, respectively. No statistically significant association was found between pregnancy status and depression severity (*χ^2^* = 4.06, *p* = 0.398).

**Table 4:** Association between pregnancy status and levels of depression and anxiety.

		Pregnancy status			
Variables	Categories	No or Unsure (%)	Yes (%)	*χ^2^* value	*p* value
Depression	Minimal	13,425 (71.30)	833 (70.50)	4.06	0.398
	Mild	4494 (23.80)	290 (24.60)		
	Moderate	690 (3.70)	48 (4.10)		
	Moderately severe	193 (1.00)	8 (0.70)		
	Severe	45 (0.20)	2 (0.20)		
Anxiety	Minimal	13,809 (73.30)	915 (77.50)	10.98	0.012*
	Mild	4212 (22.30)	217 (18.30)		
	Moderate	650 (3.40)	41 (3.50)		
	Severe	177 (0.90)	9 (0.80)		

* Significant at 5% level of significance.

#### 3.4.2 Prevalence and Severity of Anxiety by Pregnancy Status

Table 4 also presents the distribution of anxiety severity according to pregnancy status. The distribution differed significantly between pregnant and non‐pregnant women (χ^2^ = 10.98, *p* = 0.012). Pregnant women had a higher proportion of minimal anxiety (77.5% vs. 73.3%), whereas non‐pregnant women had a higher proportion of mild anxiety (22.3% vs. 18.3%). The proportions of women with moderate (3.5% vs. 3.4%) and severe anxiety (0.8% vs. 0.9%) were similar between the two groups. These findings suggest that anxiety severity was significantly associated with pregnancy status.

### 3.5 Binary Logistic Regression Analysis

The findings from the hierarchical binary logistic regression models are presented in Table 5 and Table 6.

**Table 5:** Hierarchical binary logistic regression analysis of factors associated with depression.

Variables	Model 1	Model 2	Model 3
	OR (95% CI), *p*	AOR (95% CI), *p*	AOR (95% CI), *p*
**Currently pregnant**			
No/Unsure	Ref.	Ref.	Ref.
Yes	0.99 (0.75–1.30), 0.945	1.26 (0.95–1.67), 0.106	1.29 (0.97–1.71), 0.076
**Age (years)**			
15–19	—	Ref.	Ref.
20–24	—	0.93 (0.68–1.28), 0.662	0.93 (0.68–1.28), 0.650
25–29	—	1.38 (1.02–1.87), 0.035	1.39 (1.03–1.88), 0.031
30–34	—	1.53 (1.13–2.06), 0.006	1.53 (1.13–2.06), 0.005
35–39	—	1.58 (1.17–2.13), 0.003	1.58 (1.16–2.13), 0.003
40–44	—	1.55 (1.12–2.13), 0.007	1.51 (1.09–2.08), 0.012
45–49	—	2.07 (1.50–2.86), < 0.001	1.96 (1.42–2.71), < 0.001
**Place of residence**			
Urban	—	Ref.	Ref.
Rural	—	1.07 (0.91–1.25), 0.415	1.08 (0.92–1.27), 0.325
**Educational level**			
No education	—	Ref.	Ref.
Primary	—	0.83 (0.68–1.02), 0.070	0.84 (0.69–1.03), 0.094
Secondary	—	0.88 (0.72–1.08), 0.218	0.91 (0.74–1.12), 0.368
Higher	—	0.61 (0.46–0.82), 0.001	0.64 (0.48–0.86), 0.003
**Wealth index**			
Poor	—	Ref.	Ref.
Middle	—	1.04 (0.87–1.23), 0.693	1.03 (0.87–1.22), 0.756
Rich	—	0.85 (0.72–1.01), 0.064	0.85 (0.72–1.00), 0.056
**Religion**			
Islam	—	—	Ref.
Others	—	—	0.71 (0.56–0.91), 0.006
**Marital status**			
Married	—	—	Ref.
Others	—	—	2.04 (1.63–2.55), < 0.001

**Model performance**	**Model 1**	**Model 2**	**Model 3**
Omnibus χ^2^ (*P*)	0.01 (0.945)	86.71 (< 0.001)	128.88 (< 0.001)
−2 Log Likelihood	7863.22	7776.51	7734.34
Cox & Snell *R* ^2^	0.000	0.004	0.006
Nagelkerke *R* ^2^	0.000	0.013	0.020
Hosmer–Lemeshow χ^2^ (*P*)	—	6.32 (0.611)	4.64 (0.796)

**Table 6:** Hierarchical binary logistic regression analysis of factors associated with anxiety.

Variables	Model 1	Model 2	Model 3
	OR (95% CI), *p*	AOR (95% CI), *p*	AOR (95% CI), *p*
**Currently pregnant**			
No/Unsure	Ref.	Ref.	Ref.
Yes	0.95 (0.71–1.28), 0.738	1.37 (1.01–1.86), 0.043	1.41 (1.04–1.92), 0.026
**Age (years)**			
15–19	—	Ref.	Ref.
20–24	—	1.24 (0.85–1.81), 0.271	1.23 (0.85–1.80), 0.278
25–29	—	1.63 (1.14–2.35), 0.008	1.66 (1.15–2.38), 0.007
30–34	—	2.28 (1.60–3.24), < 0.001	2.29 (1.61–3.27), < 0.001
35–39	—	2.42 (1.70–3.46), < 0.001	2.42 (1.70–3.46), < 0.001
40–44	—	2.43 (1.68–3.53), < 0.001	2.36 (1.63–3.42), < 0.001
45–49	—	2.92 (2.01–4.26), < 0.001	2.73 (1.87–3.98), < 0.001
**Place of residence**			
Urban	—	Ref.	Ref.
Rural	—	1.07 (0.91–1.26), 0.438	1.09 (0.92–1.29), 0.321
**Educational level**			
No education	—	Ref.	Ref.
Primary	—	0.91 (0.74–1.12), 0.349	0.92 (0.75–1.13), 0.431
Secondary	—	0.88 (0.72–1.09), 0.255	0.93 (0.75–1.15), 0.475
Higher	—	0.56 (0.41–0.77), < 0.001	0.60 (0.44–0.82), 0.002
**Wealth index**			
Poor	—	Ref.	Ref.
Middle	—	1.03 (0.86–1.24), 0.725	1.02 (0.85–1.23), 0.810
Rich	—	0.99 (0.83–1.18), 0.873	0.98 (0.82–1.16), 0.782
**Religion**			
Islam	—	—	Ref.
Others	—	—	0.57 (0.43–0.76), < 0.001
**Marital status**			
Married	—	—	Ref.
Others	—	—	2.41 (1.93–3.01), < 0.001

**Model performance**	**Model 1**	**Model 2**	**Model 3**
Omnibus χ^2^ (*P*)	0.11 (0.736)	112.57 (< 0.001)	181.57 (< 0.001)
−2 Log Likelihood	7195.75	7083.30	7014.30
Cox & Snell *R* ^2^	0.000	0.006	0.009
Nagelkerke *R* ^2^	0.000	0.019	0.030
Hosmer–Lemeshow χ^2^ (*P*)	—	4.71 (0.788)	12.35 (0.136)

For depression (Table 5), pregnancy status was not significantly associated with depression in any of the three models. Although the estimated odds increased after adjustment for socio‐demographic characteristics, the association remained statistically non‐significant in the fully adjusted model.

Age showed a significant association with depression. Compared with women aged 15–19 years, those aged 25–49 years had a significantly greater likelihood of experiencing depression, with the highest likelihood observed among women aged 45–49 years, who were nearly two times more likely to report depressive symptoms. Women with higher education had approximately 36% lower likelihood of depression than women with no formal education. Similarly, women belonging to religions other than Islam had about 29% lower likelihood of depression. In contrast, women who were not currently married had approximately two‐fold higher likelihood of depression than married women. Place of residence and household wealth were not significantly associated with depression.

Model performance improved after sequential adjustment. The omnibus test was statistically significant for Models 2 and 3 (*p* < 0.001), while the Hosmer–Lemeshow test indicated good model fit. The Nagelkerke R^2^ increased from 0.000 in Model 1 to 0.020 in the final model, suggesting a modest improvement in explanatory performance.

For anxiety (Table 6), pregnancy status was not associated with anxiety in the crude model. However, after adjustment for socio‐demographic characteristics, pregnant women had approximately 41% higher likelihood of anxiety than non‐pregnant women, and this association remained statistically significant in the fully adjusted model.

Age was also an important predictor of anxiety. Women aged 25–49 years had significantly higher likelihood of anxiety than those aged 15–19 years, with women aged 45–49 years showing the greatest likelihood, being approximately 2.7 times more likely to experience anxiety. Women with higher education had approximately 40% lower likelihood of anxiety than those with no education. Likewise, women from religions other than Islam had approximately 43% lower likelihood of anxiety. Women who were not currently married were more than twice as likely to experience anxiety compared with married women. No significant associations were observed for place of residence or household wealth.

The overall fit of the anxiety models improved following covariate adjustment. Both Models 2 and 3 were statistically significant according to the omnibus test (*p* < 0.001), and the Hosmer–Lemeshow test indicated adequate calibration. The Nagelkerke *R* ^2^ increased from 0.000 in Model 1 to 0.030 in the final model, indicating improved explanatory performance after adjustment.

## 4 Discussion

This study investigated the prevalence of depression and anxiety among pregnant and non‐pregnant women in Bangladesh using nationally representative data from the 2022 BDHS. Although most women reported minimal psychological symptoms, a substantial proportion experienced at least mild forms of mental health distress. Approximately 28.8% of participants reported mild‐to‐severe depressive symptoms, while 26.5% experienced mild‐to‐severe anxiety symptoms. However, when applying the standard clinical screening threshold used in the multivariable analysis (scores ≥ 10), the prevalence decreased to 4.90% for depression and 4.40% for anxiety. These findings suggest that while clinically significant depression and anxiety affected a smaller proportion of women, milder psychological symptoms were considerably more common in the population [36, 37].

The prevalence observed using the binary screening threshold is consistent with other recent analyses based on BDHS 2022 data, which similarly reported approximately 5% prevalence of clinically significant psychological distress among Bangladeshi women [38]. However, when mild symptoms are also considered, the overall burden becomes more comparable to estimates reported in South Asian studies. A recent meta‐analysis reported a pooled prevalence of antenatal depression of 24.6% across South Asia [39], which is relatively close to the combined mild‐to‐severe depressive symptom burden observed in the present study. Within the regional context, the Bangladeshi estimates remain lower than those reported in Pakistan (32.2%) and Nepal (50.0%), but are more comparable to findings from Sri Lanka (12.9%–15.9%) and India (17.7%) [39]. These variations may reflect differences in measurement tools, symptom thresholds, study populations, sociocultural settings, and healthcare access across countries

The comparatively lower estimates observed in this study may be partly explained by methodological differences across studies and the use of symptom‐based screening tools rather than diagnostic assessments. First, the application of the PHQ‐9 and GAD‐7 screening instruments measures current symptom severity, compared to the majority of regional studies with higher estimates which use diagnostic interviews or composite distress scales [38, 40]. This pattern is also evident within Bangladesh itself, where smaller‐scale studies have reported substantially higher prevalence estimates ‐ community‐based studies among rural pregnant women have documented depressive symptoms in approximately 18% and anxiety symptoms in approximately 29% of participants, while hospital‐based research has recorded even higher figures exceeding 25% for depression and 35% for anxiety. This gradient likely reflects systematic differences in study design and population characteristics, where nationally representative surveys capture a broader and potentially healthier population, whereas community and facility‐based studies tend to concentrate higher‐risk individuals. Variations in measurement tools and cutoff thresholds further contribute to these discrepancies [41, 42]. Additionally, sociocultural factors, including family support systems, may play a role, although these were not directly measured in the present study [20, 43].

This study offers a unique contribution by describing a pattern in which unadjusted and adjusted findings differ, where raw data indicate lower anxiety among pregnant women, but the adjusted estimates show the opposite. The unadjusted analysis initially suggested pregnant women scored lower on anxiety (2.77± 3.02) than non‐pregnant women (3.12 ± 3.17), but once demographic factors were adjusted for, pregnancy was associated with 41% increased odds of anxiety (AOR = 1.41, *p* = 0.026).

This pattern may reflect confounding, particularly by age and socioeconomic factors, whereby the apparent protective effect of pregnancy on anxiety is confounded by age. Younger age groups in Bangladesh are disproportionately represented among pregnant women, and tend to experience less psychological distress. So, the protective effect observed in unadjusted analyses is likely due to population structure. After adjusting for age and socioeconomic factors, pregnancy was still independently associated with greater odds of anxiety, which may be explained by pregnancy‐related stressors, such as physiological changes, anxieties about the health of the fetus, and the transition to new parenting roles. This adds to the global perinatal research literature, showing that pregnancy vulnerability may be obscured in unadjusted analyses as a result of underlying population structures [11, 44]. More importantly, this study shows the importance of using multivariable models in studies of maternal mental health, where unadjusted comparisons may mask potential risk.

On the other hand, there was no significant association between pregnancy status and depression severity (*p* = 0.398) and pregnancy was not significantly associated with depression in adjusted analyses. However, the direction of the association suggests that further investigation is warranted, particularly in longitudinal settings. This finding may suggest a lack of responsiveness of depressive symptoms to temporary physiological changes and a greater influence of stable socioeconomic and psychosocial factors such as the buffering effects of strong extended family support common in Bangladesh [20].

Model fit improved stepwise, with religion and marital status raising Nagelkerke R^2^ (0.013 to 0.020 for depression, 0.019 to 0.030 for anxiety), confirming their meaningful contribution beyond age, residence, education, and wealth. Although the Nagelkerke R^2^ values were relatively low, this is common in epidemiological and public health studies investigating complex behavioral and mental health outcomes, where psychological conditions are influenced by numerous biological, psychosocial, and environmental factors that cannot be fully captured by socio‐demographic variables alone [45, 46, 47, 48]. Therefore, low pseudo‐R^2^ values do not necessarily indicate poor model performance when the regression model demonstrates adequate calibration, statistically significant likelihood ratio tests, and clinically meaningful adjusted odds ratios. However, the low overall R^2^ indicates socio‐demographic factors alone poorly explain psychological morbidity, pointing to unmeasured factors like intimate partner violence, obstetric complications, and prior mental health history for future study.

Age and marital status were the two of the strongest predictors. Women aged 45–49 were almost three times more likely to experience anxiety (AOR = 2.73), perhaps reflecting a combination of the burdens of household work and the perimenopausal period [11, 36].

Aside from age, marital status emerged as an important psychosocial risk factor, but in the opposite direction to what is often assumed: it was women who were not currently married, rather than married women, who carried the higher burden of both depression (AOR = 2.04) and anxiety (AOR = 2.41). In the Bangladeshi context, being unmarried, whether through separation, divorce, widowhood, or never marrying, often means less spousal and in‐law support, greater financial insecurity, and social stigma in a society where marital status is closely tied to a woman's standing and security. This suggests that the absence of a stable marital support structure, rather than marriage itself, is the key psychosocial risk factor. Education, by contrast, remained protective, but only at the higher level: higher education was associated with significantly lower odds of both depression (AOR = 0.64) and anxiety (AOR = 0.60), while secondary education alone was not significant. This threshold effect likely reflects the economic independence, health literacy, and autonomy that come with advanced education [2]. Women of religions other than Islam had lower odds of depression (29%) and anxiety (43%) than Muslim women, possibly reflecting tighter social cohesion in minority communities or differential reporting of distress across cultural contexts; this warrants further qualitative research. Notably, residence and wealth were not significant predictors, suggesting distress is evenly distributed across socioeconomic strata, supporting universal rather than targeted mental health screening in maternal health services.

### 4.1 Policy Implications

The findings of this study have important implications for maternal mental health policy in Bangladesh. Since pregnancy was independently associated with a 41% higher likelihood of anxiety, routine anxiety screening should be incorporated into antenatal care services to enable early identification and timely management of women at risk. Healthcare providers should be trained to recognize symptoms of anxiety during pregnancy and ensure timely referral and appropriate psychological support when needed. As higher education was associated with lower odds of both depression and anxiety, policies that promote women's education and mental health awareness may contribute to improved psychological well‐being. Furthermore, because residence and household wealth were not significant predictors, the findings support the implementation of universal mental health screening within maternal healthcare services rather than screening based solely on socioeconomic characteristics.

### 4.2 Strengths and Limitations

This study has several important strengths. It is one of the first nationally representative studies using BDHS 2022 data to compare depression and anxiety between pregnant and non‐pregnant women in Bangladesh. The large sample size, survey‐weighted analyses, and hierarchical binary logistic regression models strengthen the robustness and generalizability of the findings. Furthermore, the sequential modeling approach allowed assessment of the independent association between pregnancy status and mental health outcomes after progressively adjusting for potential confounding factors.

Several limitations should be considered when interpreting these findings. First, the cross‐sectional design precludes causal inference, and the observed associations should not be interpreted as evidence of temporality. Second, depression and anxiety were assessed using the PHQ‐9 and GAD‐7, which, although widely validated, were not specifically designed for pregnant populations. Certain items‐ such as sleep disturbance, fatigue, and appetite changes‐ may overlap with normal physiological experiences of pregnancy, potentially leading to misclassification or overestimation of symptoms. However, the use of these instruments was determined by the BDHS survey design, and the reliance on secondary data limited the possibility of employing pregnancy‐specific tools such as the Edinburgh Postnatal Depression Scale (EPDS). Third, the relatively low proportion of pregnant women (5.90%) may have reduced statistical power for subgroup comparisons. Fourth, self‐reported screening measures, rather than clinical diagnoses, may introduce reporting bias, particularly in contexts where mental health stigma is prevalent. Finally, direct comparison with prior studies in Bangladesh is limited, as no previous nationally representative analyses have specifically examined differences in depression and anxiety between pregnant and non‐pregnant women using BDHS data.

### 4.3 Future Research

Future research should adopt longitudinal designs to better capture temporal changes in mental health across pregnancy and the postpartum period. The use of pregnancy‐specific screening instruments alongside general tools would improve measurement accuracy. Additionally, further studies are needed to explore the underlying sociocultural and healthcare‐related mechanisms influencing maternal mental health, and to validate these findings in different population subgroups and settings within Bangladesh.

## 5 Conclusion

This study provides nationally representative evidence on depression and anxiety among pregnant and non‐pregnant women in Bangladesh, showing that most women report minimal symptoms, while a notable minority experience moderate‐to‐severe condition. No significant difference was observed in depression by pregnancy status, whereas pregnancy was associated with higher odds of anxiety after adjustment. These findings highlight the importance of accounting for confounding factors when comparing mental health outcomes. Integrating routine mental health screening into maternal and reproductive health services is essential. Further longitudinal research is needed to clarify causal relationships.

## Author Contributions

**Md. Rashidul Islam Rifat:** conceptualization, methodology, writing – original draft, formal analysis. **Md. Shehab Uddin Ikhtiyer:** data curation, investigation, writing – review and editing. **Foysal Rahman Ovi:** data curation, investigation, writing – review and editing. **Md. Onthor:** writing – review and editing, validation. **Tasnia Rahman Shefa:** writing – review and editing, validation. **Most. Farjana Yesmin:** writing – review and editing, validation.

## Funding

The authors have nothing to report.

## Ethics Statement

Ethical clearance for the 2022 Bangladesh Demographic and Health Survey (BDHS) was obtained from the Institutional Review Board (IRB) of ICF International and the Bangladesh Medical Research Council (BMRC). Written informed consent was obtained from all participants prior to data collection. The present study used anonymized and publicly available secondary data obtained from the DHS Program upon formal approval; therefore, no additional ethical approval was required.

## Conflicts of Interest

The authors declare no conflicts of interest.

## Transparency Statement

Md. Rashidul Islam Rifat affirms that this manuscript is an honest, accurate, and transparent account of the study being reported; that no important aspects of the study have been omitted; and that any discrepancies from the study as planned have been explained.

## Data Availability

The data supporting the findings of this study are publicly available from the Demographic and Health Surveys (DHS) Program at https://dhsprogram.com/data/ upon registration and reasonable request. The data that support the findings of this study are available in Demographic and Health Surveys (DHS) Program at https://dhsprogram.com/data/. These data were derived from the following resources available in the public domain: ‐ Bangladesh Demographic and Health Survey (BDHS) 2022, https://dhsprogram.com/.

## References

[1] A. Larsen , J. Pintye , A. Bhat , et al., “Is There an Optimal Screening Tool for Identifying Perinatal Depression Within Clinical Settings of Sub‐Saharan Africa?,” SSM ‐ Mental Health 1 (2021): 100015, 10.1016/j.ssmmh.2021.100015. 42344140 PMC13288248

[2] M. Kamruzzaman , A. Hossain , M. A. Islam , M. S. Ahmed , E. Kabir , and M. N. Khan , “Exploring the Prevalence of Depression, Anxiety, and Stress Among University Students in Bangladesh and Their Determinants,” Clinical Epidemiology and Global Health 28 (2024): 101677. 10.1016/j.cegh.2024.101677.

[3] V. Patel , “Mental Health in Low‐ and Middle‐Income Countries,” British Medical Bulletin 81–82, no. 1 (2007): 81–96, 10.1093/bmb/ldm010. 17470476

[4] GBD 2019 Mental Disorders ., “Global, Regional, and National Burden of 12 Mental Disorders in 204 Countries and Territories, 1990–2019: A Systematic Analysis for the Global Burden of Disease Study 2019.” Lancet Psychiatry (2022). 9, 137–150. 2. 10.1016/S2215-0366(21)00395-3. 35026139 PMC8776563

[5] WHO . Depressive Disorder (Depression) [Internet]. 2025 [cited 2025 Sep 25]. https://www.who.int/news-room/fact-sheets/detail/depression.

[6] C. P. McLean , A. Asnaani , B. T. Litz , and S. G. Hofmann , “Gender Differences in Anxiety Disorders: Prevalence, Course of Illness, Comorbidity and Burden of Illness,” Journal of Psychiatric Research 45, no. 8 (2011): 1027–1035, 10.1016/j.jpsychires.2011.03.006. 21439576 PMC3135672

[7] S. Seedat , K. M. Scott , M. C. Angermeyer , et al., “Cross‐National Associations Between Gender and Mental Disorders in the World Health Organization World Mental Health Surveys,” Archives of General Psychiatry 66, no. 7 (2009): 785, 10.1001/archgenpsychiatry.2009.36. 19581570 PMC2810067

[8] Mental Health Aspects of Women's Reproductive Health [Internet]. [cited 2026 Jul 27]. https://www.who.int/publications/i/item/9789241563567.

[9] S. Jha Factors Influencing Depression and Anxiety Disorders Among Women of Reproductive Age in India – A Review of Literature [Internet]. 2022 [cited 2025 Sep 25]. https://bibalex.org/baifa/en/resources/document/476702.

[10] M. Bloch , P. J. Schmidt , M. Danaceau , J. Murphy , L. Nieman , and D. R. Rubinow , “Effects of Gonadal Steroids in Women With a History of Postpartum Depression,” American Journal of Psychiatry 157, no. 6 (2000): 924–930, 10.1176/appi.ajp.157.6.924. 10831472

[11] A. Stein , R. M. Pearson , S. H. Goodman , et al., “Effects of Perinatal Mental Disorders on the Fetus and Child,” Lancet 384, no. 9956 (2014): 1800–1819, 10.1016/S0140-6736(14)61277-0. 25455250

[12] M. Steiner , “Hormones and Mood: From Menarche to Menopause and Beyond,” Journal of Affective Disorders 74, no. 1 (2003): 67–83, 10.1016/S0165-0327(02)00432-9. 12646300

[13] C. A. Woody , A. J. Ferrari , D. J. Siskind , H. A. Whiteford , and M. G. Harris , “A Systematic Review and Meta‐Regression of the Prevalence and Incidence of Perinatal Depression,” Journal of Affective Disorders 219 (2017): 86–92, 10.1016/j.jad.2017.05.003. 28531848

[14] E. L. Lehrer , “Age at Marriage and Marital Instability: Revisiting the Becker–Landes–Michael Hypothesis,” Journal of Population Economics 21, no. 2 (2008): 463–484, 10.1007/s00148-006-0092-9.

[15] J. F. Nishat , T. E. A. Shovo , B. Ahammed , M. A. Islam , M. M. Rahman , and M. T. Hossain , “Mental Health Status of Early Married Girls During the COVID‐19 Pandemic: A Study in the Southwestern Region of Bangladesh,” Frontiers in Psychiatry 13 (2023): 1074208, 10.3389/fpsyt.2022.1074208. 36683997 PMC9849885

[16] C. E. Jordan , R. Campbell , and D. Follingstad , “Violence and Women's Mental Health: The Impact of Physical, Sexual, and Psychological Aggression,” Annual Review of Clinical Psychology 6, no. 1 (2010): 607–628, 10.1146/annurev-clinpsy-090209-151437. 20192793

[17] NIPORT . Demographic and Health Survey 2022. Dhaka, Bangladesh: National Institute of Population Research and Training (NIPORT) [Internet]. 2024 [cited 2025 Sep 26]. https://niport.gov.bd/pages/publications/bangladesh-demographic-and-health-survey-report-2022-a4a0e2-6922dad781fc96cef9eb753b.

[18] S. Rees , D. Silove , T. Chey , et al., “Lifetime Prevalence of Gender‐Based Violence in Women and the Relationship With Mental Disorders and Psychosocial Function,” Journal of the American Medical Association 306, no. 5 (2011): 513–521, 10.1001/jama.2011.1098. 21813429

[19] J. Sewalem and A. Molla , “Mental Distress and Associated Factors Among Women Who Experienced Gender Based Violence and Attending Court in South Ethiopia: A Cross‐Sectional Study,” BMC Women's Health 22, no. 1 (2022): 187, 10.1186/s12905-022-01770-6. 35597941 PMC9124378

[20] C. Dunkel Schetter , “Psychological Science on Pregnancy: Stress Processes, Biopsychosocial Models, and Emerging Research Issues,” Annual Review of Psychology 62, no. 1 (2011): 531–558, 10.1146/annurev.psych.031809.130727. 21126184

[21] K. Kroenke , R. L. Spitzer , and J. B. W. Williams , “The PHQ‐9: Validity of a Brief Depression Severity Measure,” Journal of General Internal Medicine 16, no. 9 (2001): 606–613, 10.1046/j.1525-1497.2001.016009606.x. 11556941 PMC1495268

[22] R. Banik , M. S. Islam , M. Ahmed , et al., “General Psychiatric Symptoms Among Bangladeshi People Approximately One Year After the Onset of the COVID‐19 Pandemic,” BMC Psychiatry 22, no. 1 (2022): 615, 10.1186/s12888-022-04232-3. 36123664 PMC9483885

[23] R. L. Spitzer , K. Kroenke , J. B. W. Williams , and B. Löwe , “A Brief Measure for Assessing Generalized Anxiety Disorder: The GAD‐7,” Archives of Internal Medicine 166, no. 10 (2006): 1092, 10.1001/archinte.166.10.1092. 16717171

[24] A. Kajdy , D. Sys , A. Pokropek , et al., “Risk Factors for Anxiety and Depression Among Pregnant Women During the covid ‐19 Pandemic: Results of a Web‐Based Multinational Cross‐Sectional Study,” International Journal of Gynecology & Obstetrics 160, no. 1 (2023): 167–186, 10.1002/ijgo.14388. 35932096 PMC9538861

[25] M. H. Rahman , R. M. Manna , N. G. Usmani , et al., “Depressive, Anxiety Symptoms and Their Co‐Occurrence Among Women Seeking Antenatal Care in Bangladesh,” Scientific Reports 15, no. 1 (2025): 17137, 10.1038/s41598-025-01801-w. 40382460 PMC12085565

[26] D. Wu , Q. Lin , H. Liu , et al., “The Trajectory of Depressive and Anxiety Symptom Networks Across Pregnancy: A Large‐Scale Longitudinal Study of 41,140 Women,” Journal of Affective Disorders 400 (2026): 121102, 10.1016/j.jad.2025.121102. 41520842

[27] WHO . Mental Disorders [Internet]. 2022 [cited 2025 Sep 25]. https://www.who.int/news-room/fact-sheets/detail/mental-disorders.

[28] H. S. Alamri , A. Algarni , S. F. Shehata , et al., “Prevalence of Depression, Anxiety, and Stress Among the General Population in Saudi Arabia During Covid‐19 Pandemic,” International Journal of Environmental Research and Public Health 17, no. 24 (2020): 9183, 10.3390/ijerph17249183. 33316900 PMC7764434

[29] R. Sandal , N. Goel , M. Sharma , R. Bakshi , N. Singh , and D. Kumar , “Prevalence of Depression, Anxiety and Stress Among School Going Adolescent in Chandigarh,” Journal of Family Medicine and Primary Care 6, no. 2 (2017): 405, 10.4103/2249-4863.219988. PMC5749094 29302555

[30] M. T. Hasan , T. Anwar , E. Christopher , et al., “The Current State of Mental Healthcare in Bangladesh: Part 1 – An Updated Country Profile,” BJPsych International 18, no. 4 (2021 Nov): 78–82, 10.1192/bji.2021.41. 34747942 PMC8554893

[31] DHS Program . Bangladesh Standard DHS 2022 Dataset [Internet]. 2022. https://dhsprogram.com/data/dataset/Bangladesh_Standard-DHS_2022.cfm.

[32] M. R. Kanchon , J. Sani , T. Ahmed , M. R. Akon , and F. M. Aliyu , “Interpretable Machine Learning for Predicting Early Mental Health Care‐Seeking Among Reproductive‐Age Women in Bangladesh Using BDHS 2022 Data,” Discover Public Health 23, no. 1 (2026): 1039, 10.1186/s12982-026-02344-9.

[33] M. T. Amin , T. Ara , B. Pal , et al., “Prevalence and Correlates of Anxiety and Depression Among Ever‐Married Reproductive‐Aged Women in Bangladesh: National‐Level Insights From the 2022 Bangladesh Demographic and Health Survey,” BMC Public Health 25, no. 1 (2025 Mar 26): 1143, 10.1186/s12889-025-22228-y. 40133877 PMC11938691

[34] A. Mimi , “Prevalence and Determinants of Common Mental Health Illnesses Among Reproductive‐Aged Women in Bangladesh: Evidence From Demographic and Health Surveys Data 2022. Bhusal S, Editor,” PLOS Ment Health 3, no. 2 (2026): e0000352, 10.1371/journal.pmen.0000352. 41712565 PMC12919789

[35] S. Esteban‐Gonzalo , M. H. A. Khan , M. M. Hasan , and J. F. Díaz‐Morales , “Psychosocial Predictors of Women's Mental Health in Bangladesh: Evidence From BDHS‐2022 Data on Fertility, Empowerment, Decision‐Making, Child Loss, and Sex‐Affective Behaviours,” Public Health 254 (2026): 106222, 10.1016/j.puhe.2026.106222. 41795247

[36] M. R. Haque , A. M. Fazle Rabbi , F. Araf , and M. M. Rahman , “Prevalence of Anxiety and Depression Among Married Women in Bangladesh: An analysis of Nationally Representative Survey,” PLOS Mental Health 22 7 (2025): e0000387. 10.1371/journal.pmen.0000387. PMC12798323 41662041

[37] S. Raza , R. Banik , S. T. A. Noor , et al., “Anxiety and Depression Among Reproductive‐Aged Women in Bangladesh: Burden, Determinants, and Care‐Seeking Practices Based on a Nationally Representative Demographic and Health Survey,” Archives of Women's Mental Health 28, no. 5 (2025): 1125–1141, 10.1007/s00737-025-01564-3. PMC12436553 39964560

[38] M. F. Alam , H. U. Ahmed , M. T. Alam , et al., “Community Prevalence of Psychiatric Disorders: Findings From a Nationwide Survey in Bangladesh,” Asian Journal of Psychiatry 92 (2024): 103897, 10.1016/j.ajp.2023.103897. 38199203

[39] R. Mahendran , S. Puthussery , and M. Amalan , “Prevalence of Antenatal Depression in South Asia: A Systematic Review and Meta‐Analysis,” European Journal of Public Health 30, no. Suppl_5 (2020): ckaa165.557, 10.1093/eurpub/ckaa165.557. 31010821

[40] H. H. Annajigowda , L. P. Nirisha , S. Ganjekar , et al., “Common Mental Disorders Among Women in Reproductive Age Group: An Analysis of National Mental Health Survey, India 2016,” Indian Journal of Psychiatry 65, no. 12 (2023): 1238–1243, 10.4103/indianjpsychiatry.indianjpsychiatry_832_23. 38298883 PMC10826868

[41] H. E. Nasreen , Z. N. Kabir , Y. Forsell , and M. Edhborg , “Prevalence and Associated Factors of Depressive and Anxiety Symptoms During Pregnancy: A Population Based Study in Rural Bangladesh,” BMC Women's Health 11, no. 1 (2011): 22, 10.1186/1472-6874-11-22. 21635722 PMC3117808

[42] N. R. Mahin , M. J. Hossain , B. Bhowmick , and L. R. Kundu , “Investigating the Prevalence and Associated Factors of Anxiety and Depressive Symptoms Among Pregnant Women in Bangladesh: A Hospital‐Based Cross‐Sectional Study,” Health Science Reports 8, no. 8 (2025): e71144, 10.1002/hsr2.71144. 40772115 PMC12326430

[43] E. R. Severinsen , L. K. A. Kähler , S. E. Thomassen , et al., “Mental Health Indicators in Pregnant Women Compared With Women in the General Population During the Coronavirus Disease 2019 Pandemic in Denmark,” Acta Obstetricia et Gynecologica Scandinavica 100, no. 11 (2021): 2009–2018, 10.1111/aogs.14258. 34546563 PMC8653239

[44] N. Insan , A. Weke , S. Forrest , and J. Rankin , “Social Determinants of Antenatal Depression and Anxiety Among Women in South Asia: A Systematic Review & Meta‐analysis,” PLOS ONE 17 2 (2022): e0263760. 10.1371/journal.pone.0263760. 35139136 PMC8827460

[45] A. O'Neill , S. Gallagher , A. Hannigan , and K. Robinson , “Association Between Work Status and Depression in Informal Caregivers: A Collaborative Modelling Approach,” European Journal of Public Health 32, no. 1 (2022): 59–65, 10.1093/eurpub/ckab178. 34849725 PMC8807115

[46] B. Gavurova , V. Ivankova , M. Rigelsky , T. Mudarri , and M. Miovsky , “Somatic Symptoms, Anxiety, and Depression Among College Students in the Czech Republic and Slovakia: A Cross‐Sectional Study,” Frontiers in Public Health 10 (2022): 859107, 10.3389/fpubh.2022.859107. 35359763 PMC8961809

[47] F. Méndez‐López , B. Oliván‐Blázquez , M. Domínguez‐García , et al., “Depression and Anxiety Symptoms, Health Behavior–Related Personal Factors, and Tobacco Smoking Problem: A Secondary Data Analysis,” Frontiers in Public Health 14 (2026): 1886431, 10.3389/fpubh.2026.1886431.

[48] X. Liu , W. Zheng , L. Hoon , et al., “Predictors of Depression Outcomes Among University Students Following Brief Smartphone‐Based Interventions,” NPJ Mental Health Research 5, no. 1 (2026): 25, 10.1038/s44184-026-00208-3. 41998112 PMC13090371

